# GP-Plotter: Flexible Spectral Visualization for Proteomics Data with Emphasis on Glycoproteomics Analysis

**DOI:** 10.1093/gpbjnl/qzae069

**Published:** 2024-10-08

**Authors:** Zheng Fang, Mingming Dong, Hongqiang Qin, Mingliang Ye

**Affiliations:** State Key Laboratory of Medical Proteomics, National Chromatographic R. & A. Center, CAS Key Laboratory of Separation Science for Analytical Chemistry, Dalian Institute of Chemical Physics, Chinese Academy of Sciences, Dalian 116023, China; School of Bioengineering, Dalian University of Technology, Dalian 116024, China; State Key Laboratory of Medical Proteomics, National Chromatographic R. & A. Center, CAS Key Laboratory of Separation Science for Analytical Chemistry, Dalian Institute of Chemical Physics, Chinese Academy of Sciences, Dalian 116023, China; University of Chinese Academy of Sciences, Beijing 100049, China; State Key Laboratory of Medical Proteomics, National Chromatographic R. & A. Center, CAS Key Laboratory of Separation Science for Analytical Chemistry, Dalian Institute of Chemical Physics, Chinese Academy of Sciences, Dalian 116023, China; University of Chinese Academy of Sciences, Beijing 100049, China

**Keywords:** Proteomics, Glycosylation, Glycoproteomics, Software, Visualization

## Abstract

Identification evaluation and result dissemination are essential components in mass spectrometry-based proteomics analysis. The visualization of fragment ions in mass spectrum provides strong evidence for peptide identification and modification localization. Here, we present an easy-to-use tool, named GP-Plotter, for ion annotation of tandem mass spectra and corresponding image output. Identification result files of common searching tools in the community and user-customized files are supported as input of GP-Plotter. Multiple display modes and parameter customization can be achieved in GP-Plotter to present annotated spectra of interest. Different image formats, especially vector graphic formats, are available for image generation which is favorable for data publication. Notably, GP-Plotter is also well-suited for the visualization and evaluation of glycopeptide spectrum assignments with comprehensive annotation of glycan fragment ions. With a user-friendly graphical interface, GP-Plotter is expected to be a universal visualization tool for the community. GP-Plotter has been implemented in the latest version of Glyco-Decipher (v1.0.4) and the standalone GP-Plotter software is also freely available at https://github.com/DICP-1809.

## Introduction

Mass spectrometry (MS)-based proteomics has become mainstream in large-scale identification and localization of post-translational modifications (PTMs), including complex protein glycosylation [1–3]. A number of identification software tools are developed to provide peptide assignments for the acquired tandem mass spectra [4–6]. With the fast development of enrichment methods [7–9] and MS instruments [10–12], many large-scale glycoproteomic datasets have been collected, which facilitates the study of biological functions of protein glycosylation and the discovery of novel disease biomarkers. To interpret glycopeptide spectra, several software tools with diverse search strategies, including Byonic [13], pGlyco3 [14], MSFragger-Glyco [15], GPQuest [16], and our Glyco-Decipher [17], are developed. However, as illustrated in a recent glycoproteomics community study [18], discrepant glycopeptide results are often reported for the same datasets. Inspection of identification results by visualizing and comparing annotated glycopeptide spectra is a crucial part for the evaluation of glycopeptide assignment.

The visualization of fragment ions in tandem mass spectra is important for manual validation of peptide identification and modification localization. And enormous advances in data visualization have been achieved in proteomics bioinformatics [19–22]. Nevertheless, existing proteomics tools are not applicable for glycopeptide spectrum annotation due to their inability in glycan ion matching. Currently, a few glycoproteomics software tools support glycopeptide spectrum visualization, but there are still limitations in use. For example, Byonic [13] provides a glycopeptide spectrum viewer in graphical user interface (GUI) and provides image output for annotated glycopeptide spectra. However, due to the lack of structure information in glycan database, Byonic only matches Y fragment ions of glycan core structure, leaving Y ions with complex compositions unannotated (Figure S1A). pGlyco [14] incorporates a plugin named gLabel to provide spectrum annotation and image output. Pixel-based graphics file format (*i.e.*, png) images with fixed parameters are generated by gLabel (Figure S1B), posing potential restrictions in customization and publication. More importantly, these software tools only support the visualization of glycopeptide spectra based on their own identification results, which hinders comparison and assessment across identification pipelines. Universal visualization tools for identification results from diverse identification tools are still lacking for the community.

**Figure 1:**
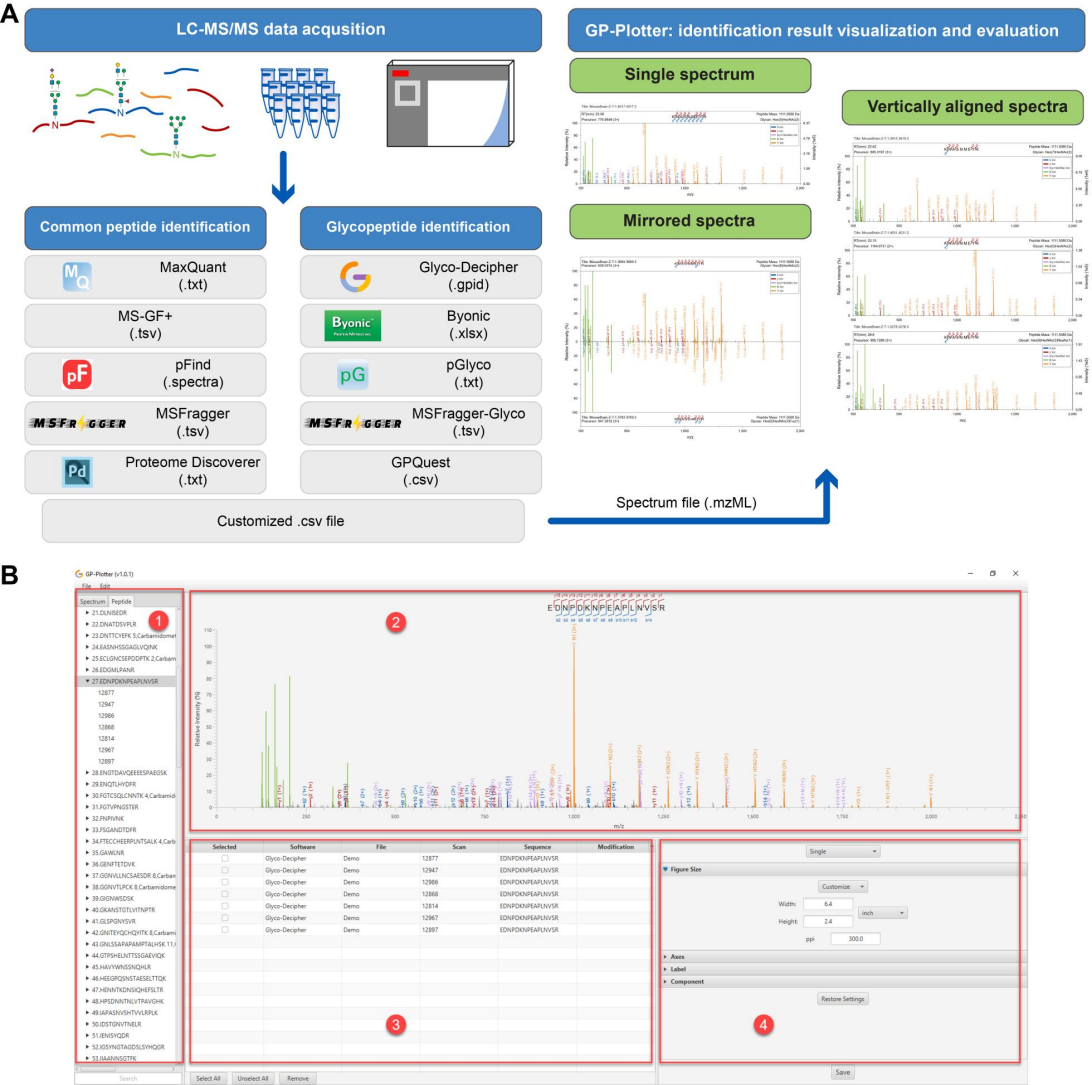
The overall design of GP-Plotter **A**. Overview of GP-Plotter. **B**. Main graphic interface of GP-Plotter for users. The main panel of GP-Plotter contains four parts. (1) List of peptide identifications and corresponding mass spectra which are indexed with spectrum scan number. (2) Visualization pane of the selected spectrum in the spectrum identification list. (3) Table of candidate mass spectra to be saved as image. (4) Parameter setting pane for image generation.

Here, to facilitate identification inspection and spectral visualization, we present a user-friendly tool named GP-Plotter for displaying and outputting annotated tandem mass spectra of proteomics (**Figure 1**). GP-Plotter enables fragment ion annotation for diverse modified peptides based on the output result of common identification tools. Especially, it enables glycan ion annotation for intact glycopeptide spectra. As a flexible tool, GP-Plotter can also annotate acquired MS data encoded in open mzML format according to user-customized parameters. Multiple display modes for annotated spectra are supported in GP-Plotter, and several key parameters in image generation could be easily customized by users via graphic interface. Additionally, common image file formats, including vector graphics, are supported in GP-Plotter. GP-Plotter has been implemented in the latest version of Glyco-Decipher (v1.0.4), and the standalone GP-Plotter executable program is freely available at https://github.com/DICP-1809.

## Method

### Implementation of GP-Plotter

GP-Plotter is coded in Java11 (v11.0.11) and Python 3.9 (v3.9.13). Python packages of matplotlib (v3.5.2) and seaborn (v0.11.2) are utilized for spectrum image generation. GP-Plotter can be run as a standalone executable program in GUI mode, and it has also been implemented in the latest version of Glyco-Decipher (v1.0.4). Spectrum file in mzML [23] format, which is open source and could be easily obtained by format converters (*e.g.*, MSConvert [24]), is adopted in GP-Plotter as spectrum input. Identification result files of common proteomics tools (*e.g.*, MaxQuant, MS-GF+, and MSFragger) and glycoproteomics tools (*e.g.*, Glyco-Decipher, Byonic, pGlyco3, MSFragger-Glyco, and GPQuest), are supported and could be directly used as input of GP-Plotter. To extend the application of GP-Plotter, users can also provide self-customized .csv file, which contains columns of scan number, peptide, and optional glycan information, as input of GP-Plotter to view tandem mass spectra of interest. For modifications not in Unimod database, users can provide self-defined modifications used in peptide identification. The modification information in configuration file is split by “\t” tab and consists of four parts: (1) modification name; (2) modification mass; (3) modification chemical composition; and (4) modification sites and neutral losses.

### Enumeration of theoretical peptide and glycan fragment ions

The type of fragment ions generated by peptides is highly dependent on the dissociation method(s) adopted in data acquisition. Visualization of mass spectra generated by collisional activation [including collision-induced dissociation (CID) and higher-energy collisional dissociation (HCD)], electron-driven dissociation [*e.g.*, electron-transfer dissociation (ETD) and electron-capture dissociation (ECD)], and hybrid activation [combination of ETD and CID (ETciD) and combination of ETD and HCD (EThcD)] methods is supported in GP-Plotter, in which corresponding types of theoretical peptide fragments are enumerated and matched with experimental data. More importantly, the annotation of glycan fragment ions in glycopeptide spectra is also crucial for the evaluation of glycan assignment in glycosylation analysis. With structure information of glycans, an enumeration algorithm, which aims to list all possible glycan B/Y ions, is embedded in GP-Plotter. GP-Plotter supports glycan structure information encoded in WURCS [25] and GlycoCT [26] formats. These formats are widely used in glycomics studies and can be easily obtained in common glycan databases (*e.g.*, GlyTouCan [27]). After the enumeration of theoretical B/Y ions, glycan ions are matched with glycopeptide spectra generated by collisional activation methods, which induce dissociation of highly labile glycosidic bonds.

### Spectrum annotation and image output

After ion matching between theoretical fragments and experimental data with user-defined mass tolerance, annotated tandem mass spectra are displayed in the panel of GP-Plotter and could be added into candidate table in GUI panel for subsequent image output. Users can adjust the parameters of the annotated spectrum images. The modifiable parameters include image size, axis range, and labeled ion information. Three modes are supported in GP-Plotter for image generation, including (1) single spectrum mode, which presents a single mass spectrum in one image; (2) mirrored spectrum mode, in which two spectra that share the x-axis are displayed in one image, with one spectrum is shown upward and the other is shown downward; (3) vertically aligned spectrum mode, in which multiple tandem mass spectra are displayed vertically with aligned boundaries. In addition to pixel-based raster graphics formats, vector graphics file formats, including .svg, .pdf, and .eps, are also supported for image generation to facilitate publication of annotated spectrum results.

### Availability of GP-Plotter

All functions of GP-Plotter can be invoked after the installation of Java (v11.0.11 or higher) and Python (v3.9.X, X ≥ 13) environments in Windows operating system. In addition to the standalone version, GP-Plotter has also been incorporated into Glyco-Decipher (since v1.0.4), to facilitate result evaluation and image generation after glycopeptide identification. The integrated GP-Plotter can be found in the tool list at the GUI of Glyco-Decipher.

## Results

### GP-Plotter supports flexible file input and image output

GP-Plotter is designed for the ion annotation of tandem mass spectra and corresponding image generation. It supports result files of a variety of proteomics identification software tools (Figure S2). In addition, users can also import customized .csv files to view annotated spectra of interest, which enables the visualization of identification results from other identification tools in the community. With spectrum data in standard open mzML format, GP-Plotter can provide universal ion annotation and spectral visualization for the community of proteomics (Figure 1A). Many key parameters in image generation (*e.g.*, image size, axis range, and ion label) could be customized via the GUI of GP-Plotter, which facilitates identification evaluation and result publication (Figure S3). In addition to displaying single spectrum in one image, mirrored/vertically aligned spectra make the discrepancy between spectra conspicuously and improve efficiency in identification inspection with GP-Plotter. The flexible parameter setting and image output make GP-Plotter a promising proteomics data visualization software over other existing tools (Figure S4). Next, we used several examples to demonstrate the advantages of GP-Plotter.

### GP-Plotter enables comprehensive annotation of both peptide and glycan fragment ions

**Figure 2:**
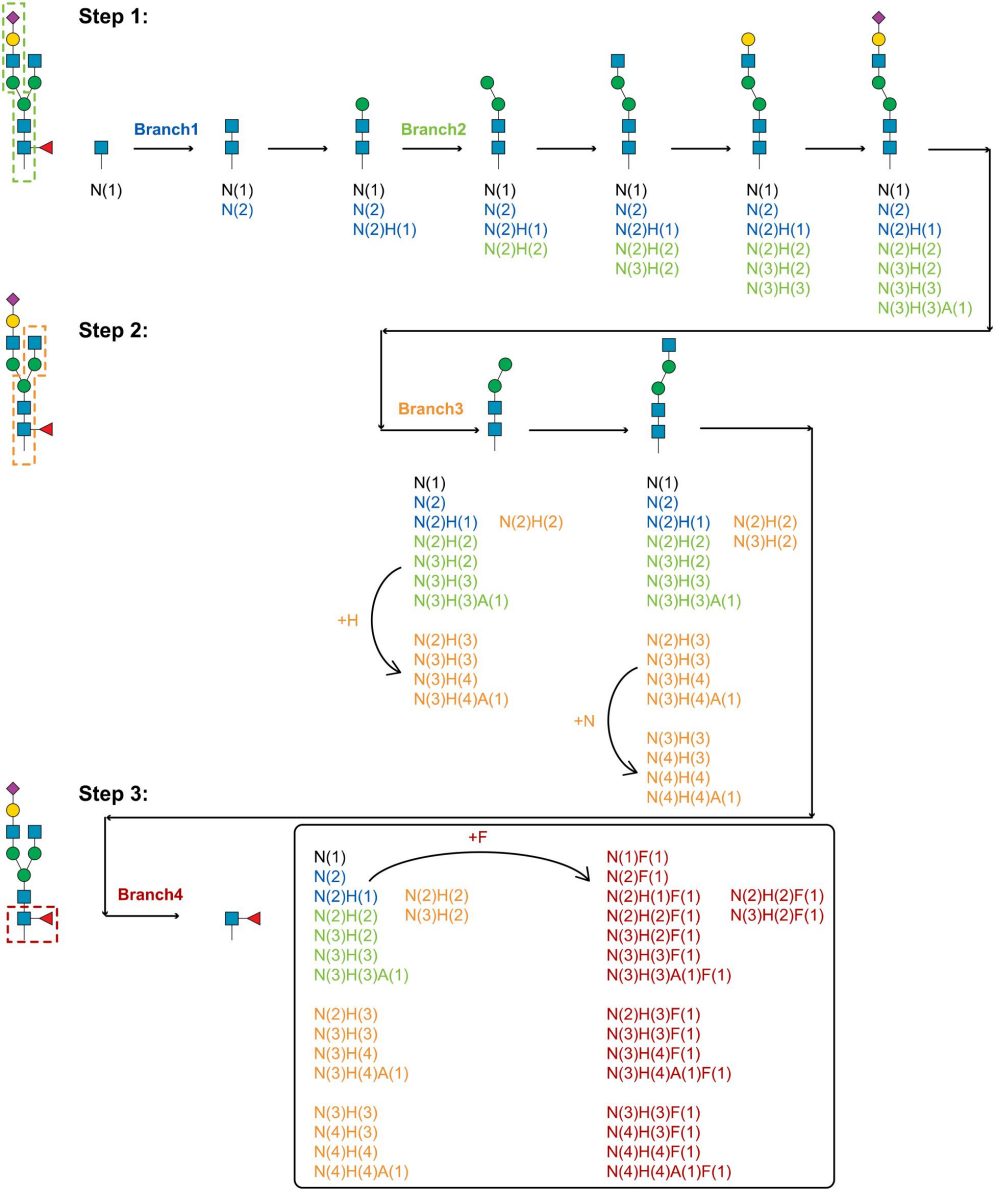
The enumeration algorithm for theoretical glycan Y fragment ions in glycopeptide spectrum annotation H: Hex, hexose; N: HexNAc, *N*-acetylhexosamine; A: NeuAc, *N*-acetyl-neuraminic acid; F: Fuc, fucose.

Unlike the linear structure of peptides, the tree structure of glycans poses challenges in fragment enumeration and spectrum annotation. The algorithm principle for glycan ion enumeration in GP-Plotter is stepping all branches of the glycan structure, which is exemplified in **Figure 2** with glycan of Hex(4)HexNAc(4)NeuAc(1)Fuc(1). GP-Plotter starts at the root saccharide node of a glycan and steps toward the terminal saccharide node of a branch (Step 1 in Figure 2). And corresponding fragment ions are enumerated when stepping each monosaccharide in the branch. After reaching to the terminal of a branch, GP-Plotter returns to the nearest junction node of two branches (hexose of core structure in this example) and steps in the other branch (Step 2 in Figure 2). In addition to glycan ions for the new branch, corresponding monosaccharide is also added to glycan ions of the before branch, which aims to incorporate all theoretical fragment ions for branch structures. Then, the process is performed for all branch structures in a glycan (Step 3 in Figure 2). Finally, glycan Y ions with duplicate composition are removed. All theoretical glycan B ions are also enumerated by the direction-reversed stepping algorithm. After adding with peptide mass, theoretical glycan Y ions, glycan B ions, and common oxonium ions (Table S1) are matched to experimental glycopeptide spectra. As exemplified in Figure S5, by annotating spectra with glycan ions incorporated with structure information, GP-Plotter is of great beneficial for diagnostic ion inspection and glycan structural analysis.

### GP-Plotter facilitates evaluation of spectrum interpretation across different identification tools

**Figure 3:**
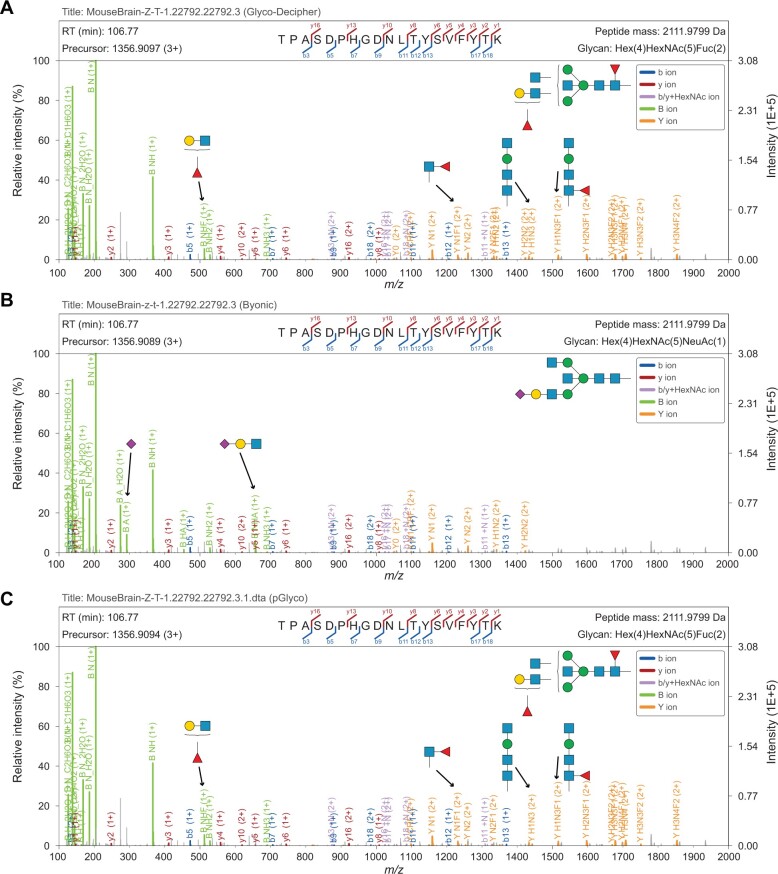
Close inspection of chimeric spectra identified by different software tools with ion annotation of GP-Plotter **A**. Spectrum annotation based on the result of Glyco-Decipher. **B**. Spectrum annotation based on the result of Byonic. **C**. Spectrum annotation based on the result of pGlyco 3.0. In the glycopeptide spectrum, the identical peptide sequence of “TPASDPHGDNLTYSVFYTK” was identified by Glyco-Decipher, Byonic, and pGlyco3, but different glycan compositions were matched due to the isotopic shift in precursor correction: Hex(4)HexNAc(5)Fuc(2) was matched by Glyco-Decipher and pGlyco3, but Hex(4)HexNAc(5)NeuAc(1) was matched by Byonic.

Combined with mirrored/vertically aligned spectrum mode in GP-Plotter, evaluation of spectrum interpretation provided by different identification tools can be easily achieved. **Figure 3** presents a glycopeptide spectrum as an example, in which identical peptide backbone “TPASDPHGDNLTYSVFYTK” was identified by Glyco-Decipher, Byonic, and pGlyco. However, different glycan compositions were matched by the three tools due to the isotopic shift in precursor correction: “Hex(4)HexNAc(5)Fuc(2)” was matched in Glyco-Decipher and pGlyco, and “Hex(4)HexNAc(5)NeuAc(1)” was matched in Byonic. After careful analysis with annotated spectral visualization provided by GP-Plotter, the glycopeptide spectrum is more likely to be a chimeric one on account of close mass values of the two glycans (Mass_Fuc(2)_ – Mass_NeuAc(1)_ = 1 Da) and corresponding diagnostic fragment ions in MS2. Furthermore, GP-Plotter can assist the evaluation of identification confidence when discrepancy results are obtained. For example, in comparison with Glyco-Decipher and pGlyco, Byonic reported a different glycan result for the glycopeptide spectrum shown in Figure S6. And the absence of diagnostic oxonium ion (NeuGc, *m*/*z* = 308.098) among the fragment ions of the glycopeptide indicates that the glycan assignment of Byonic might be a random match. Given universal supports for the file formats of identification software tools, GP-Plotter is expected to be a visualization tool widely used in result assessment and confidence evaluation for glycoproteomics analysis.

### GP-Plotter is a universal tool for the visualization and verification of modification assignment

**Figure 4:**
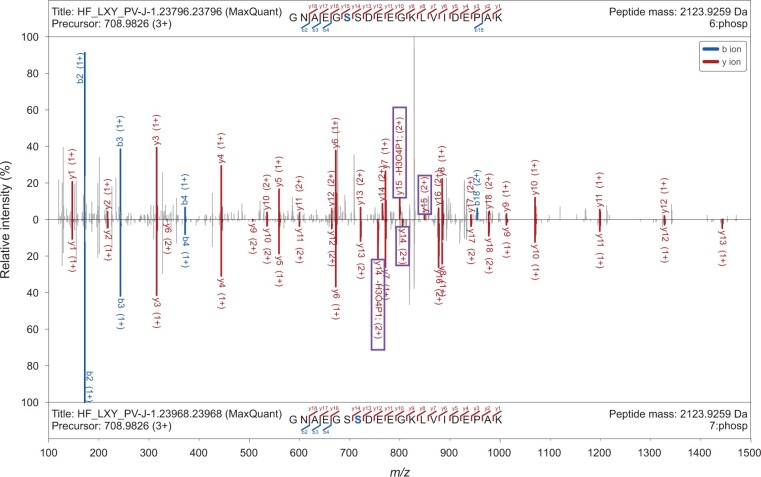
Spectral visualization in GP-Plotter assists modification site localization on peptide sequences Diagnostic fragment ions, including neutral loss ions, for different phosphorylation sites (S6 and S7) on the peptide “GNAEGSSDEEGKLVIDEPAK” can be easily detected in the mirrored spectrum mode provided by GP-Plotter.

In functional study of PTMs, manual verification is an essential step after identification and localization of modification on peptides, especially for those with multiple potential sites. A typical example is phosphorylation, which mainly happens at serine (S), threonine (T), and tyrosine (Y) residues in proteins [28]. Diagnostic fragment ions with phosphorylated residues are of great value for the determination of modification sites and could be evident in the spectral visualization of GP-Plotter. For example, S132 and S133 in the protein hepatoma-derived growth factor (UniProt: P51858) are two potential phosphorylation sites annotated by UniProt database. By visualizing MS data acquired in a recent study [29], supporting diagnostic fragment ions (including neutral loss ions) of peptides with phosphorylated S132 and S133, respectively, are clearly shown by the mirrored spectrum mode of GP-Plotter despite the high spectral similarity (**Figure 4**). Supports for glycopeptide spectra of ETxxD (ETD, EThcd, and ETciD) fragmentation also facilitate the verification of glycosite localization in glycoproteomics analysis, especially for *O*-glycosylation (Figure S7). The abovementioned examples demonstrate the potential broad applications of GP-Plotter in proteomics analysis.

## Discussion

Evaluating the performance of ion matching between tandem mass spectra and modified peptides is crucial for assessing the accuracy of site-specific modification assignments. The high micro-heterogeneity of glycosylation and the diversity of glycan structures largely increase the complexity of fragment ions in glycopeptide spectra. A number of identification strategies are developed to decipher large-scale glycoproteomic datasets [13–15,30], yet the visualization of glycopeptide spectra is still lagging behind compared to proteomics bioinformatics development. Apart from peptide ions, the fragment ions of glycan part are also considered to be crucial information in glycan structure discrimination and false discovery rate control [14]. To address the issue of data visualization, we developed GP-Plotter, an easy-to-use tool to provide mass spectrum annotation and image output. Both peptide ions and glycan ions are comprehensively annotated by GP-Plotter, enabling evaluation of the mass spectrum assignment reported by different glycoproteomics identification software tools. However, there are still limitations in GP-Plotter at current stage: (1) glycan ions of cross ring fragmentation, which are useful in glycomics analysis, are rare in glycopeptide spectrum and are not considered in GP-Plotter; (2) TIMS-TOF data cannot be processed in GP-Plotter because of additional ion mobility dimension and spectrum combination step. Overall, GP-Plotter supports result files of a variety of proteomics identification tools, making it a useful visualization tool in modification analysis. The adjustment of plotting parameters and display modes enable users to generate their customized images. With the ability to output pixel/vector graphics, GP-Plotter makes MS data visually accessible and accelerates the preparation of figures for publication.

## Supplementary Material

qzae069_Supplementary_Data

## Data Availability

GP-Plotter is available at https://github.com/DICP-1809.

## CRediT author statement

**Zheng Fang:** Conceptualization, Software, Visualization, Writing – original draft. **Mingming Dong:** Supervision. **Hongqiang Qin:** Supervision. **Mingliang Ye:** Project administration, Funding acquisition, Supervision, Writing – review & editing. All authors have read and approved the final manuscript.
