# Supplementary material for: GP-Plotter: Flexible Spectral Visualization for Proteomics Data with Emphasis on Glycoproteomics Analysis
Source: Genomics Proteomics Bioinformatics. 2024 Oct 8;22(5):qzae069. doi: 10.1093/gpbjnl/qzae069 (PMC11661977; doi:10.1093/gpbjnl/qzae069)
Supplement: qzae069_Supplementary_Data [file qzae069_supplementary_data.zip › supplementary material captions.docx]

**Supplementary material**

**Figure S1 Examples of annotated spectra generated by (A) Byonic and (B) pGlyco**

**Figure S2 Input and output file formats supported in GP-Plotter**

**A.** Software identification result files supported in GP-Plotter as inputs. **B.** Formats of spectrum image that can be generated by GP-Plotter.

**Figure S3 Parameters that could be customized by users in GP-Plotter for image output**

As a user-friendly software tool, GP-Plotter enables several key parameters that could be customized by users to generate informative images of annotated tandem mass spectra. The modifiable parameters include: 1) Mass tolerance in ion matching; 2) Figure size (width and height); 3) Margins around glycopeptide spectrum (top, bottom, left and right); 4) Axis Labels; 5) Ranges of axis (start and end values of x/y-axis); 6) Color and width of lines for matched ions; 7) Color and size of labels for matched ions; 8) Optional information of labeled ions (ion type, charge, *m*/*z* and intensity); 9) Header information of mass spectra; 10) Legend of matched ions.

**Figure S4 Features of GP-Plotter in comparison with other existing data visualization tools**

**Figure S5 Discrimination of isobaric structures of glycan could be achieved in GP-Plotter**

**A.** Mass spectra of glycopeptides with the same peptide backbone (“KNASNMEYR”) and glycan composition (“Hex(5)HexNAc(4)Fuc(1)”), but are different in glycan structures. Possible structure illustrations for the glycans are manually added in the figure. **B.** High *m*/*z* section of the glycopeptide spectra shown in (a) by setting the x-axis range to 600-2000 in GP-Plotter. The diagnostic Y ions for isobaric structure discrimination are labeled in the figure.

**Figure S6 GP-Plotter facilitates the evaluation of glycopeptide identification provided by different software tools**

**A.** Glyco-Decipher; **B.** Byonic; **C.** pGlyco 3.0.

**Figure S7 Visualization of HCD-pd-EThcD glycopeptide spectrum in GP-Plotter helps the validation of glycosite localization**

Annotated O-glycopeptide spectra based on the identification and site localization of pGlyco3. The dataset was downloaded from PRIDE with accession PXD037415 [1]. Raw file “SS_VVA_05062022_EThcD.raw” and corresponding identification result “SS_VVA_05062022_EThcD.txt” were loaded into GP-Plotter without modification.

**A.** HCD spectrum with scan 6215 and **B.** EThcd spectrum with scan 6217 were identified with glycopeptide “RVPSEAPPTEVPDRDPEK S4,HexNAc(1); T9, HexNAc(1); 18,Guanidinyl[K];”, and were selected and outputted. Instead of glycosylation with a single glycan of HexNAc(2), the peptide is glycosylated with two single HexNAc(1) glycans: the observation of c3, c4 and y/z14, z15 validates the HexNAc(1) glycosylzation at S4. The observation of c8, b/c9 validates another HexNAc(1) glycosylzation at T9. [1], Suttapitugsakul S, Matsumoto Y, Aryal RP, Cummings RD. Large-scale and site-specific mapping of the murine brain O-glycoproteome with IMPa. Analytical Chemistry 2023;95:13423–30.

**Table S1 Oxonium ions matched in GP-Plotter**
