# Supplementary material for: GP-Plotter: Flexible Spectral Visualization for Proteomics Data with Emphasis on Glycoproteomics Analysis
Source: Genomics Proteomics Bioinformatics. 2024 Oct 8;22(5):qzae069. doi: 10.1093/gpbjnl/qzae069 (PMC11661977; doi:10.1093/gpbjnl/qzae069)
Supplement: qzae069_Supplementary_Data [file qzae069_supplementary_data.zip › Figure S4.pdf]

		Skyline	PeptideShaker	Byonic	pGlyco	GP-Plotter
Input	Universal File Input	✓	✓	✗	✗	✓
Spectrum Annotation	*m*/*z* Range Customization	✗	✓	✓	✗	✓
	Annotation of Complex Glycan Fragments	✗	✗	✗	✓	✓
	Label Color/Size Customization	✗	✓	✗	✗	✓
Image Output	Image Size Customization	✗	✓	✓	✗	✓
	Mirrored and Vertial Aligned Spectra	✗	✗	✗	✗	✓
	Vector Graphics Format	✓	✓	✓	✗	✓
