# Supplementary material for: GP-Plotter: Flexible Spectral Visualization for Proteomics Data with Emphasis on Glycoproteomics Analysis
Source: Genomics Proteomics Bioinformatics. 2024 Oct 8;22(5):qzae069. doi: 10.1093/gpbjnl/qzae069 (PMC11661977; doi:10.1093/gpbjnl/qzae069)
Supplement: qzae069_Supplementary_Data [file qzae069_supplementary_data.zip › Figure S2.pdf]

A

Software	File	Essential Information Column
**Common Proteomics Software**		
**MaxQuant**	msms.txt	Raw file, Scan number, m/z, Charge, Sequence, Modifications, Modified sequence
**MSFragger**	psm.tsv	Spectrum, Calculated Peptide Mass, Calibrated Observed M/Z, Charge, Observed Mass, Peptide, Assigned Modifications, Observed Modifications
**MS-GF+**	[File].tsv	#SpecFile, ScanNum, Peptide, Precursor, Charge
**pFind**	pFind.spectra	File_Name, Scan_No, Charge, Exp.MH+, Sequence, Modification, Q-value, Target/Decoy
**Proteome Discoverer (Mascot/Sequest Node)**	RESULTS_PSMs.txt	Spectrum File, First Scan, Sequence, Modifications, mz in Da, Charge
**Customized CSV**	[File].csv	File, Scan, Peptide, Modification
**Common Glyco-Proteomics Software**		
**Glyco-Decipher**	[File].gpid	XML Format File
**Byonic**	[File].xlsx	Peptide<ProteinMetrics Confidential>, Glycans NHFAGNa, Protein Name, Scan #, Observed m/z, z
**MSFragger-Glyco**	psm.tsv	Spectrum, Calibrated Observed M/Z, Charge, Observed Mass, Peptide, Assigned Modifications, Observed Modifications
**pGlyco**	pGlycoDB-GP-FDR-Pro.txt	RawName, Scan, Charge, PrecursorMZ, Peptide, Mod, GlycanComposition, PlausibleStruct
**GPQuest**	[File].csv	MS2, RT, Precursor MZ, Precursor Charge, Sequence, Peptide, glycan
**Customized CSV**	[File].csv	File, Scan, Peptide, Modification, Glycan

B

Format	Description
.png	Portable Network Graphics
.pdf	Portable Document Format
.eps	Encapsulated Postscript
.jpeg	Joint Photographic Experts Group
.jpg	Joint Photographic Experts Group
.pgf	PGF code for LaTeX
.ps	Postscript
.raw	Raw RGBA bitmap
.rgba	Raw RGBA bitmap
.svg	Scalable Vector Graphics
.svgz	Scalable Vector Graphics
.tif	Tagged Image File Format
.tiff	Tagged Image File Format
