# Supplementary figures and images for: GP-Plotter: Flexible Spectral Visualization for Proteomics Data with Emphasis on Glycoproteomics Analysis
Source: Genomics Proteomics Bioinformatics. 2024 Oct 8;22(5):qzae069. doi: 10.1093/gpbjnl/qzae069 (PMC11661977; doi:10.1093/gpbjnl/qzae069)

**A**

SS\_VVA\_05062022\_ETHcD.6215.6215.3 (pGlyco3)

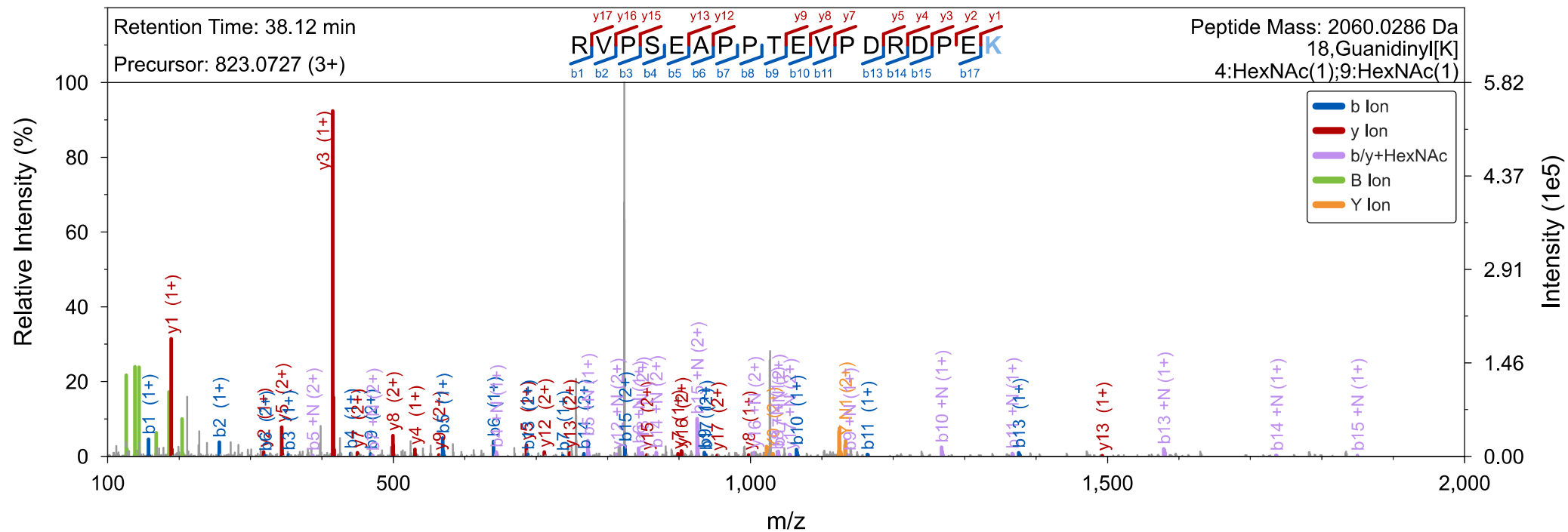**B**

SS\_VVA\_05062022\_ETHcD.6217.6217.3 (pGlyco3)

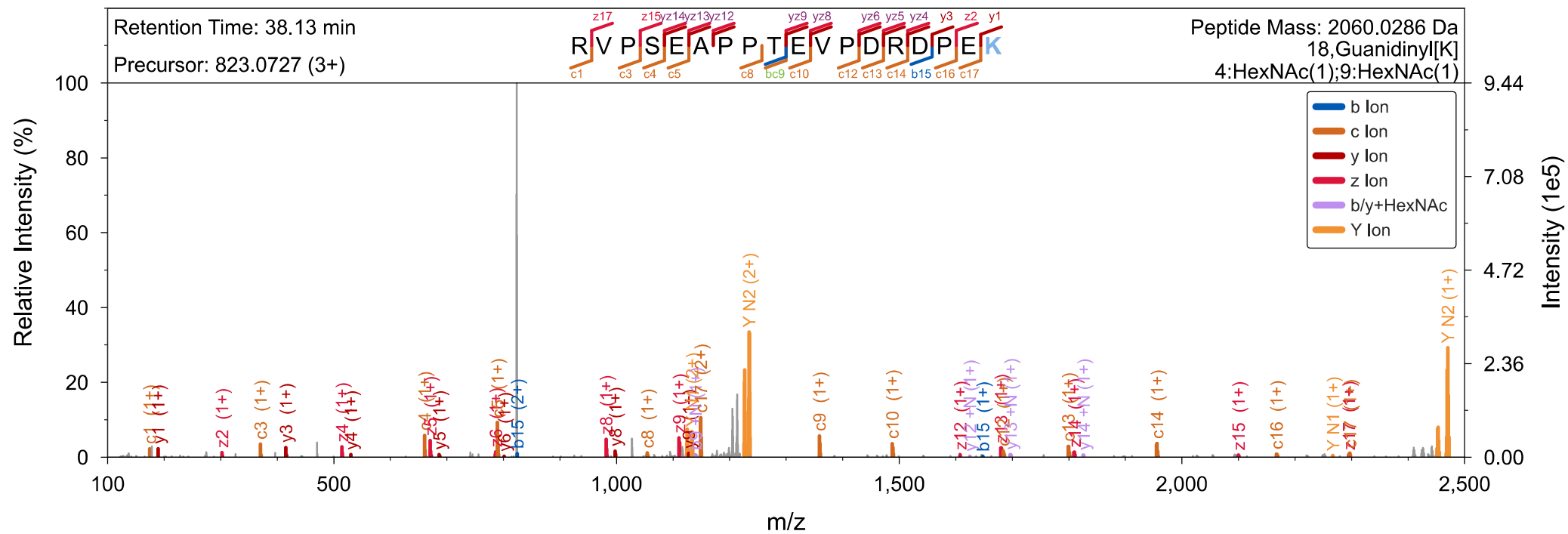

Supplement: qzae069_Supplementary_Data [file qzae069_supplementary_data.zip › Figure S7.pdf]

A

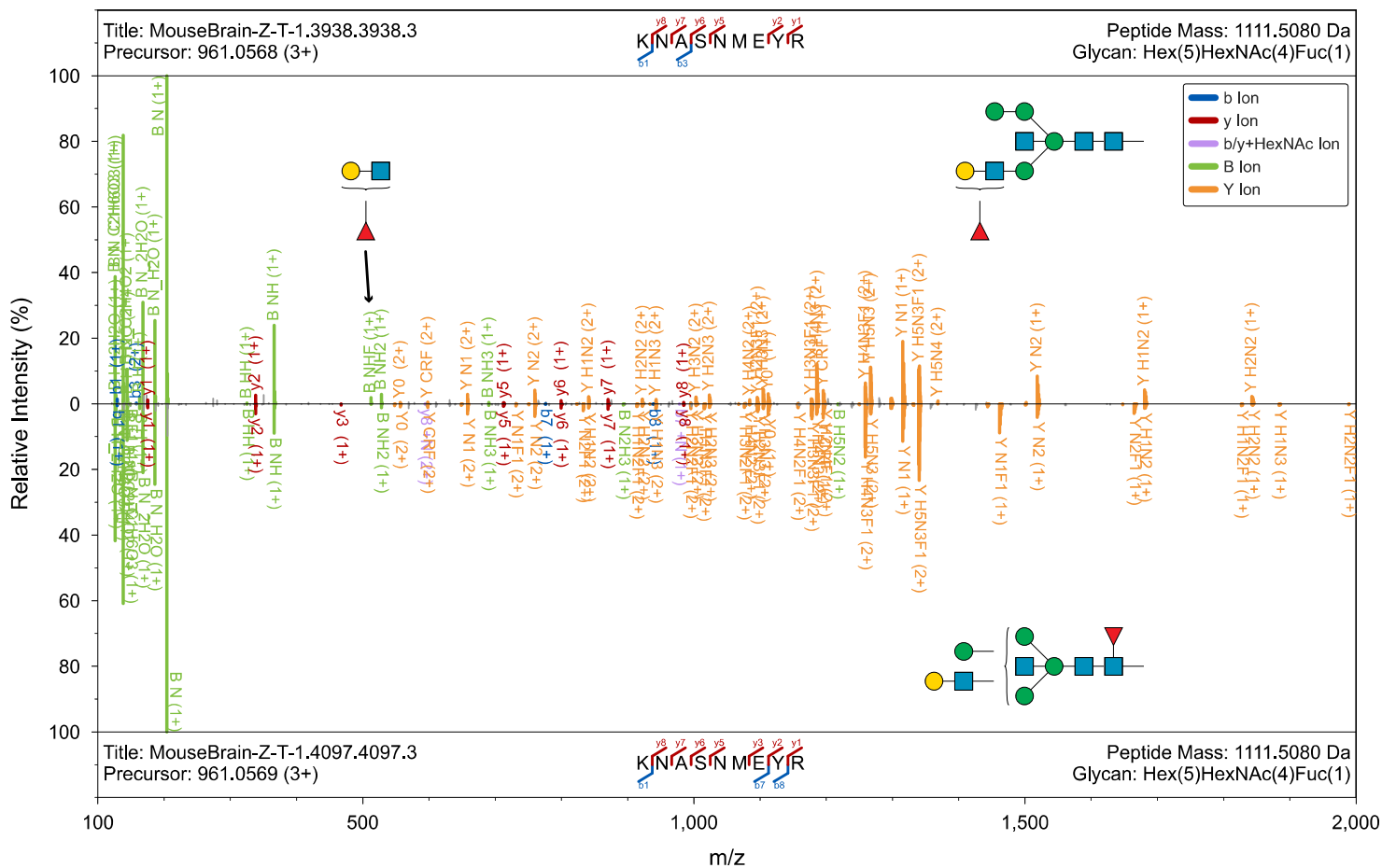

B

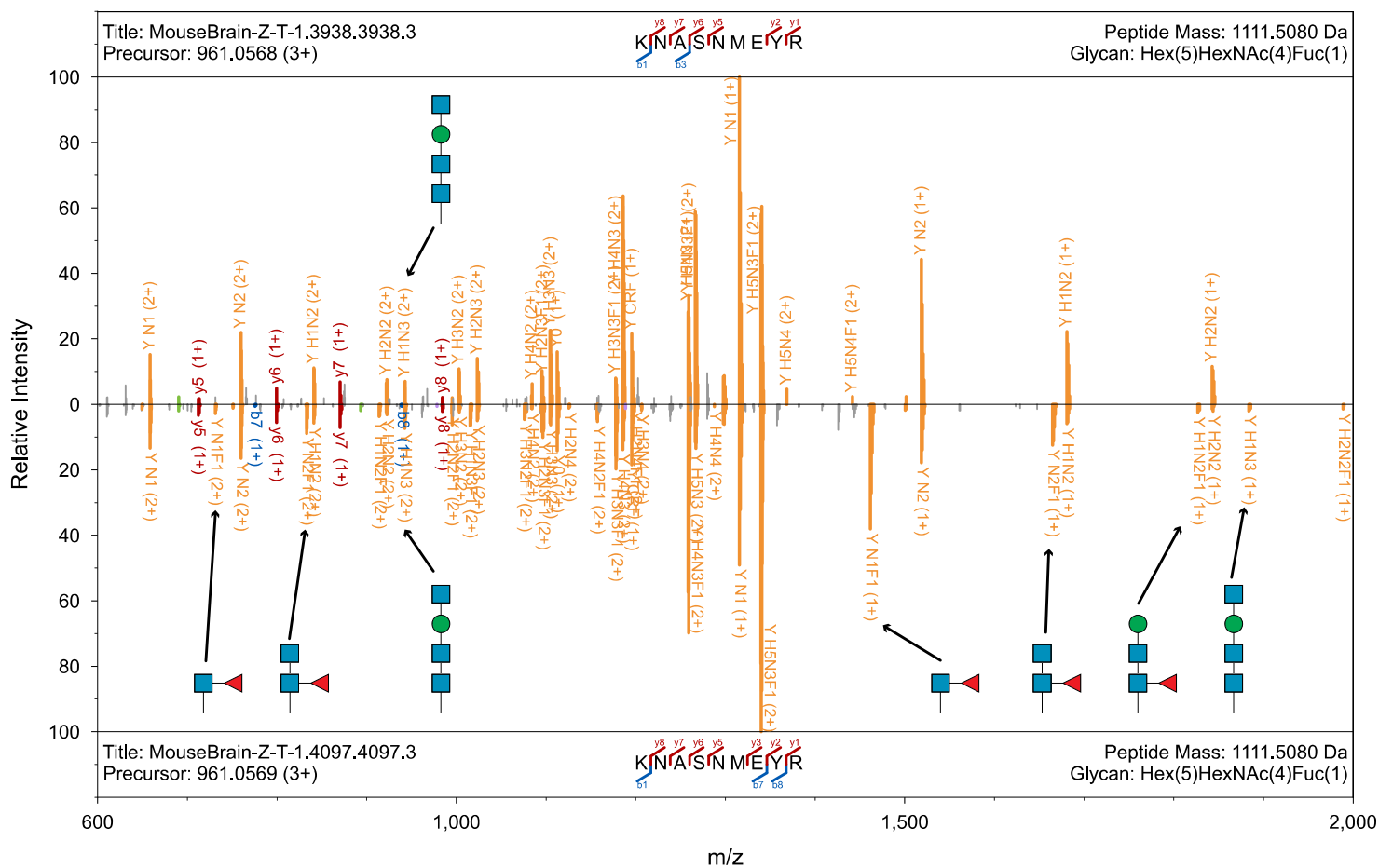

Supplement: qzae069_Supplementary_Data [file qzae069_supplementary_data.zip › Figure S5.pdf]

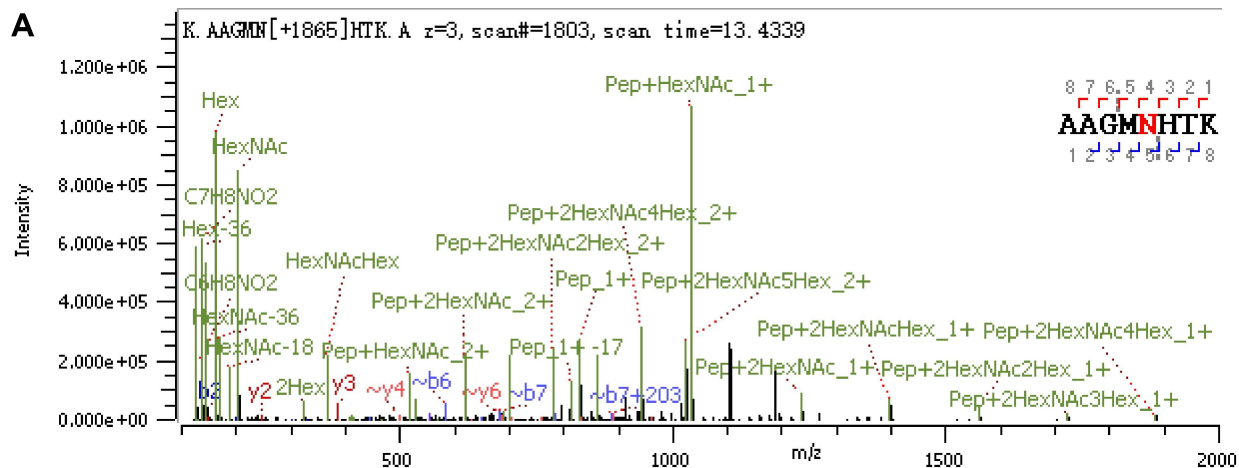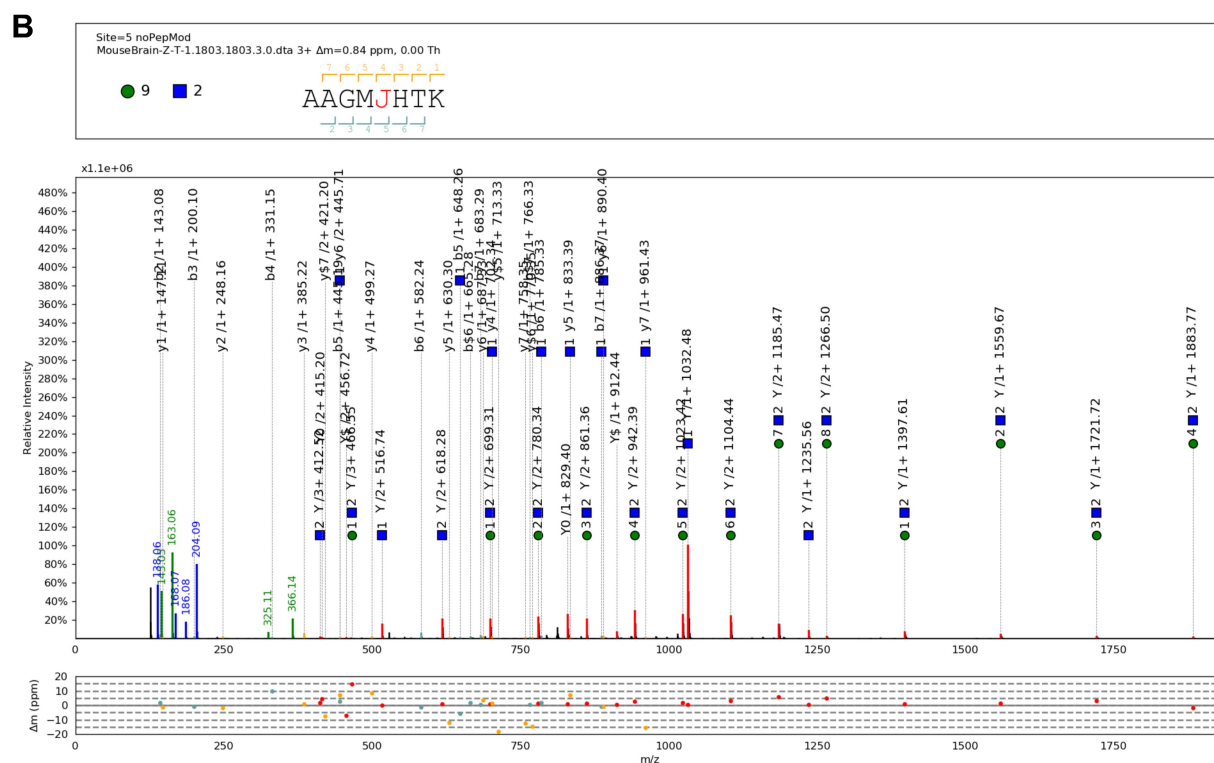

Supplement: qzae069_Supplementary_Data [file qzae069_supplementary_data.zip › Figure S1.pdf]

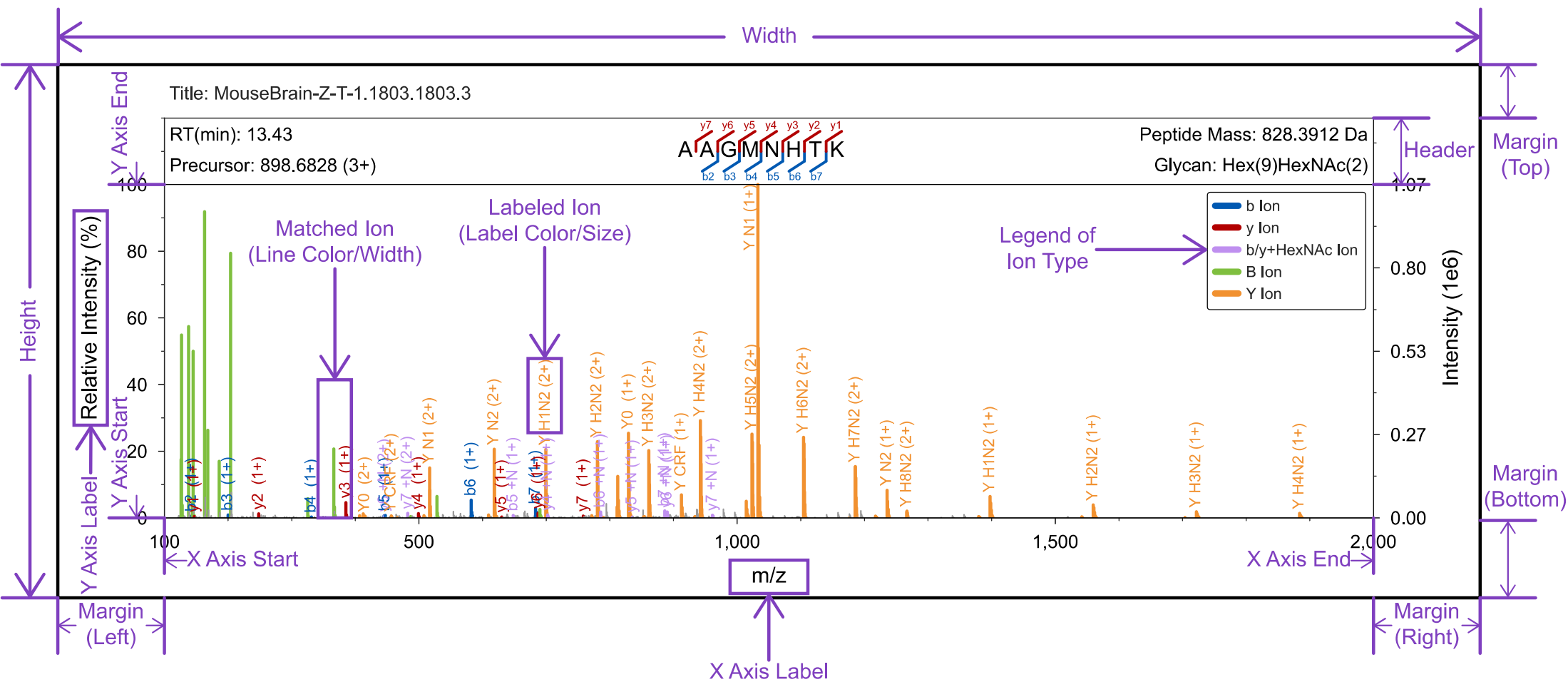

Supplement: qzae069_Supplementary_Data [file qzae069_supplementary_data.zip › Figure S3.pdf]

**A**

Title: MouseBrain-Z-T-1.12975.12975.3

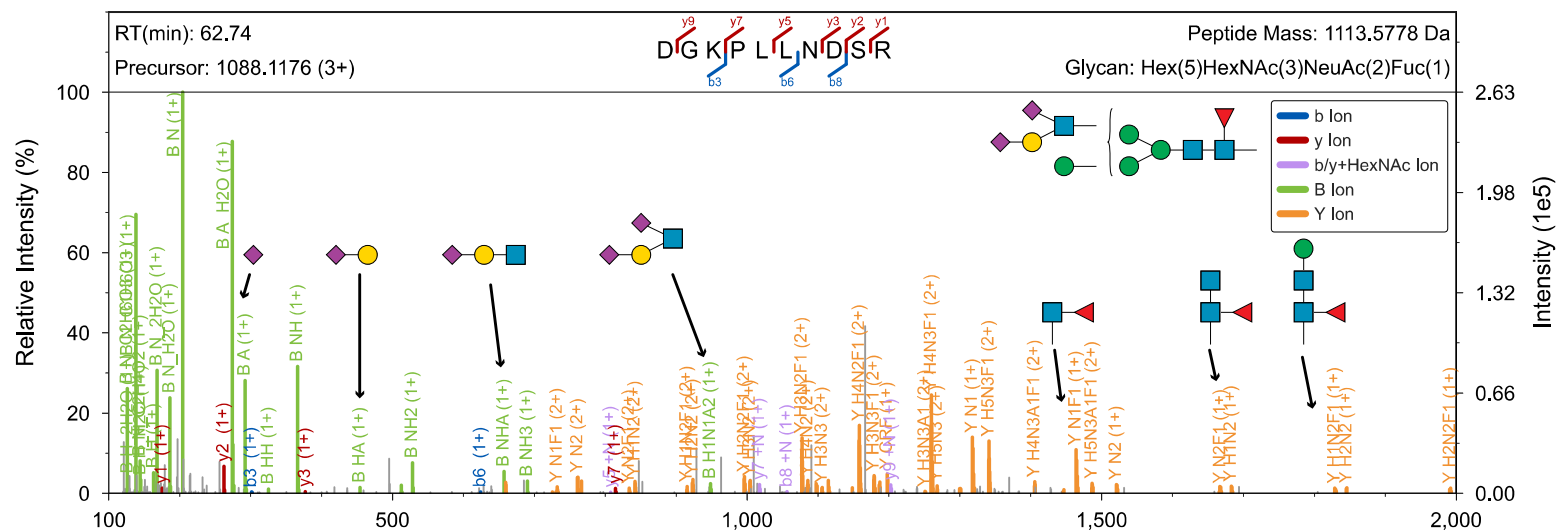**B**

Title: mousebrain-z-t-1.12975.12975.3 (Byonic)

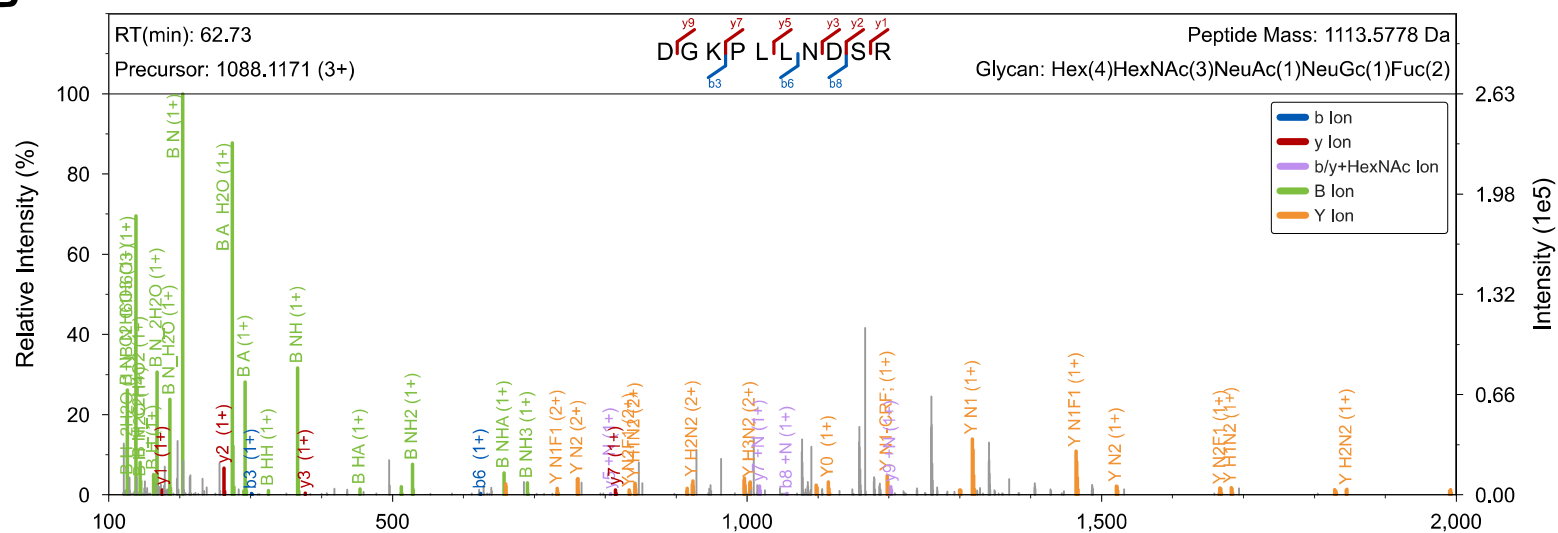**C**

Title: MouseBrain-Z-T-1.12975.12975.3.0.dta (pGlyco)

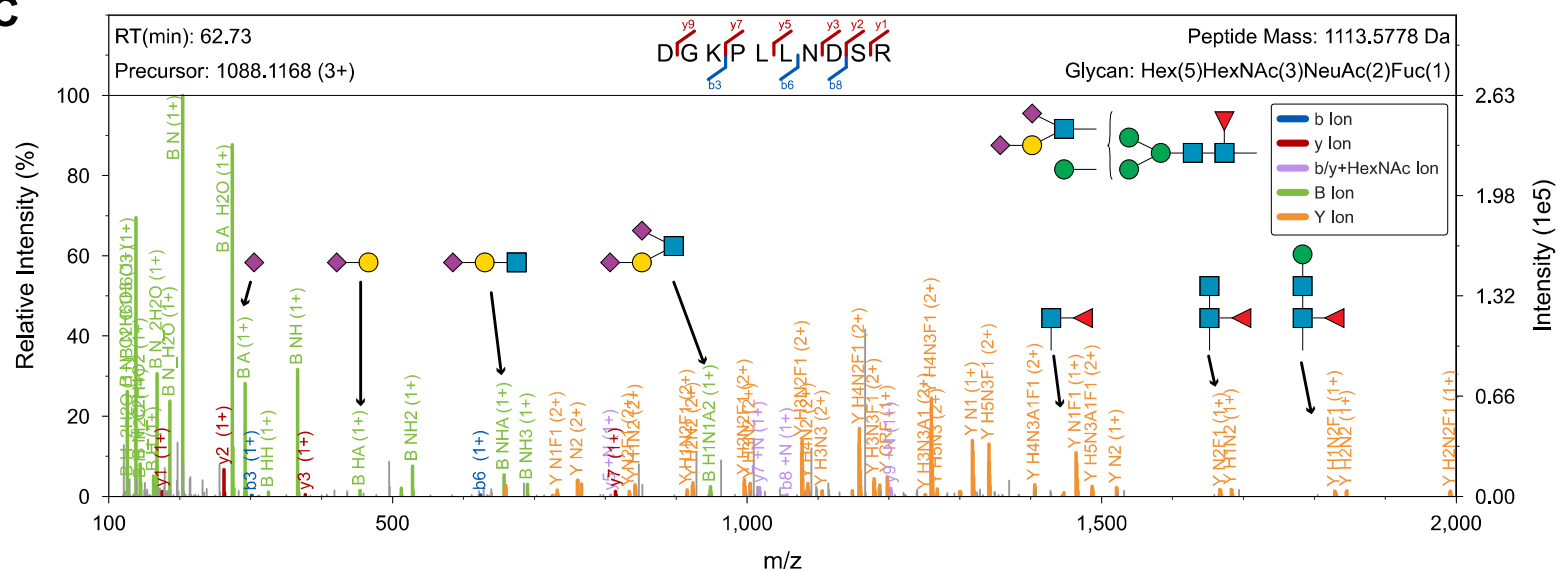

Supplement: qzae069_Supplementary_Data [file qzae069_supplementary_data.zip › Figure S6.pdf]
